# Using Client Reminders to Increase Colorectal Cancer Screening in Montana, 2012

**DOI:** 10.5888/pcd11.130274

**Published:** 2014-04-03

**Authors:** Lisa Troyer, Laura L. Williamson, Leah Merchant, Eugene J. Lengerich

**Affiliations:** Author Affiliations: Laura L. Williamson, Leah Merchant, Montana Department of Public Health and Human Services, Helena, Montana; Eugene J. Lengerich, Department of Public Health Sciences, Penn State University, Penn State Hershey Cancer Institute, Hershey, Pennsylvania.

## Abstract

**Background:**

Colorectal cancer (CRC) is the third leading cause of cancer death for men and women in the United States. CRC screening can save lives by detecting precancerous polyps that are then removed or by detecting cancer early when treatment is most effective.

**Community Context:**

CRC screening participation in Montana is low. To increase screening participation among Montanans with health insurance, the Montana Cancer Control Programs (MCCP) partnered with a small association health organization (AHO). This partnership implemented a postcard campaign to increase CRC screening participation among the AHO’s enrollees.

**Methods:**

Postcards were sent to 1,011 people insured through the AHO; 504 people were mailed 1 postcard and 507 people were mailed 2 postcards. Evaluation of the campaign assessed recall of the campaign among people who received 1 postcard versus people who received 2 postcards.

**Outcome:**

Women were 60% more likely to recall receiving the postcards than were men. People who received 2 postcards were 2.3 times as likely to recall receiving them as were people who received 1 postcard.

**Interpretation:**

The MCCP considers this collaborative project with an AHO a promising approach to implementing evidence-based colorectal cancer screening interventions. The MCCP plans to partner with additional AHOs in Montana to evaluate CRC screening participation among their enrollees.

## Background

Colorectal cancer (CRC) is the third leading cause of cancer death among men and women in the United States (1). Screening for CRC can save lives by detecting precancerous polyps so that they can be removed before developing into cancer or by detecting cancer early when treatment is the most effective. The US Preventive Services Task Force (Task Force) recommends screening for CRC via fecal occult blood testing, sigmoidoscopy, or colonoscopy for average risk adults starting at age 50 years and continuing until age 75 years (2). The Centers for Disease Control and Prevention (CDC) estimates that if everyone in the United States 50 years or older had appropriate screening tests, approximately 60% of CRC deaths could be prevented (3). Current CRC screening rates in the United States are low; in 2010, only 65% of US adults aged 50 to 75 years reported having appropriate CRC screening (4). However, in 2010, only 61% of Montanans with health care coverage reported being up-to-date with CRC screening (5). The *Healthy People 2020* goal is to have 70.5% of adults up-to-date on CRC screening based on the most recent guidelines (6).

To address the low rate of CRC screening in the United States, the Task Force reviewed the literature and found strong evidence to recommend the use of small media, such as postcards and other mailers, to increase use of the fecal occult blood test (FOBT) for CRC screening (7). However, the Task Force found insufficient evidence to determine the effectiveness of a postcard campaign to increase CRC screening by flexible sigmoidoscopy, colonoscopy, or double contrast barium enema because no studies evaluating these screening procedures were found (7).

## Community Context

Historically, CRC screening participation has been low in Montana. In 2006, Montana’s Behavioral Risk Factor Surveillance System survey (BRFSS) found that only 53% of adults aged 50 years or older reported ever having had a colonoscopy or sigmoidoscopy (5). Because adults in a rural state like Montana may have unique barriers to CRC screening, such as long travel distances or cost that would limit access to health care, the Montana Cancer Control Programs (MCCP) sought to identify potential barriers to CRC screening for Montana residents. According to state-added questions in the 2007 Montana BRFSS, the leading reasons for not being screened were respondents’ beliefs that it was not necessary, no health care provider recommended the procedure, or the cost was high (8). Distance to a facility was not identified as a barrier. The MCCP performed a Colonoscopy Screening Capacity Survey in 2008 to estimate the maximum annual screening colonoscopy capacity of communities with colonoscopy facilities. This survey found that Montana had the capacity to meet increased demand for colonoscopy screening and that it would be feasible to implement population-based interventions to increase screening colonoscopy (9). In 2009, the MCCP received funding from the CDC Colorectal Cancer Control Program to provide direct CRC screening services to low-income men and women and to implement population-based approaches to increase CRC screenings in Montana. This shift from individual one-on-one screening recommendations to a systems-based approach was intended to increase CRC screenings.

Still, in 2010, only 61% of Montanans aged 50 years or older with health care coverage reported being up-to-date with CRC screening (7). Consequently, the MCCP sought to increase CRC screening among Montanans with health insurance through a systems-based approach.

To increase CRC screening among insured Montanans, the MCCP approached the major insurance carriers in Montana to maximize reach throughout the state. The MCCP made multiple unsuccessful attempts to meet with decision makers in the large insurance companies in Montana to collaborate and gain approval for an outreach campaign and to collect claims data. Consequently, the MCCP pursued organizations that were either self-insured or had a large group insurance benefit plan.

 MCCP first approached insurance organizations before CRC screening was covered as a preventive benefit on insurance plans through the Affordable Care Act (ACA). Because the ACA required that recommended cancer screenings, including CRC screening, be covered as a preventive benefit in all US health insurance plans, the MCCP adjusted its strategy. One insurance carrier provided insurance benefits to Medicare beneficiaries and was willing to partner with MCCP to promote CRC screenings to increase their Healthcare Effectiveness Data and Information Set (HEDIS) rates. After using a postcard campaign with Medicare recipients, the MCCP began pursuing small association health organizations (AHOs) that were either insured with the larger insurance companies in Montana or self-insured to conduct outreach on the benefits of preventive cancer screenings, including colorectal, breast, and cervical cancer screenings. For the purpose of this article, an example of an AHO is the Montana Association of Counties Health Care Trust, a health care self-insured pool. These types of AHOs were receptive to partnership activities. In establishing this partnership, the MCCP offered to pay the costs of this outreach campaign, including postage and printing costs.

In 2011, the MCCP began working with an AHO that insures approximately 1,800 county government workers throughout Montana; approximately 1,000 of those workers are aged 50 through 75 years. Current employees and spouses were covered. Insured employees older than 65 are secondary payers to Medicare.

The objectives of this project were to 1) partner with an AHO to increase CRC screening participation among its enrollees and 2) assess the effectiveness of a small-media campaign to increase CRC screening among adults with health care coverage in Montana. The intended outcomes for the MCCP were 1) to develop an ongoing relationship with an AHO to implement evidence-based, statewide cancer screening interventions, 2) assess if recall of small-media campaigns increases with the receipt of 2 pieces of small media, and 3) increase the AHO members’ use of CRC insurance benefits.

## Methods

In accordance with the Task Force’s guidelines, the MCCP implemented a small-media campaign to increase use of the CRC screening benefits available through the AHO’s insurance benefit plan. The campaign consisted of mailing postcards and placing 2 articles in AHO newsletters on cancer incidence and cancers that are detectable through screening.

Through the National Cancer Institute’s Research to Reality Mentorship Program between MCCP employee and mentee Lisa Troyer, BA, and mentor Eugene Lengerich, VMD, MS, short- and long-term evaluation indicators were developed for working with insurance organizations. As a result of this mentorship, the MCCP implemented a new intervention consisting of postcards and surveys to determine whether AHO members recalled receiving the postcards and if they took action after receiving one. The action of getting a CRC screening would then be reflected in insurance claims data. Evaluation of this small media assessed whether recipients recalled receiving a postcard from the MCCP and AHO about getting screened for CRC.

**Figure 1:**
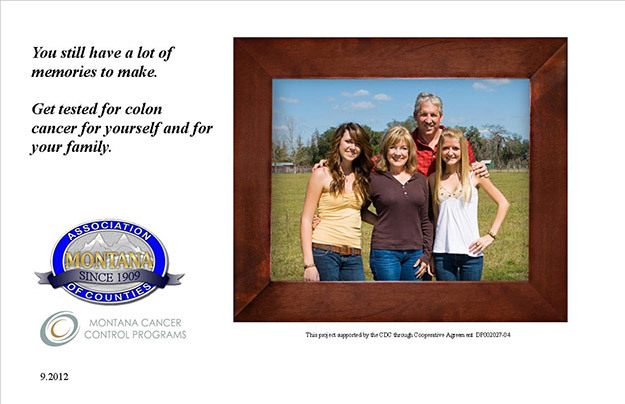
Image of first postcard mailed to intervention groups A and B in September 2012, Montana.

**Figure 2:**
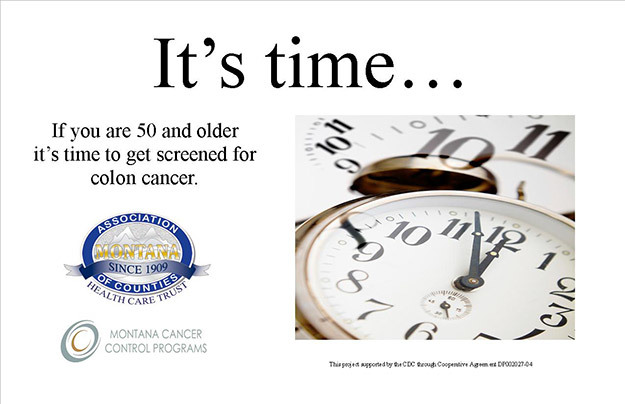
Image of second postcard mailed only to intervention group B in October 2012, Montana.

The AHO identified all enrollees aged 50 to 75 years (n = 1,011) and randomly assigned them to 1 of 2 groups according to county of residence. Group A included people from counties beginning with the letters A through G, and Group B included people from counties beginning with the letters H through Z. Group A comprised 14 counties, representing 51 towns and 504 enrollees. Group B comprised 23 counties, representing 68 towns and 507 enrollees. The AHO wanted all enrollees to receive a minimum of 1 postcard for CRC education. Group A was mailed 1 postcard in September 2012; Group B was mailed a first postcard in September 2012 and a second postcard 3 weeks later in October 2012. The postcards that both intervention groups received were identical. However, Group B’s second postcard had a different image and message (Figures 1 and 2).

The MCCP used Make It Your Own (MIYO) for the development of the message that was introduced to the AHO. MIYO was created in 2008 by the Health Communication Research Laboratory at Washington University in St. Louis. MIYO is a Web-based system that gives community partners the tools to create customized, culturally appropriate, health-related materials targeted to their audience with audience-tested images and messages (www.MIYOworks.org).

The MCCP provided multiple postcard examples to the AHO, and the AHO chose the image and message. A major criterion for the organization was an image that resonated with the demographics of their insured population, predominantly white families living in rural Montana communities. The back side of both postcards listed insurance coverage information pertaining to the health benefits plan, stating the preventive cancer screening benefits covered and listing the colorectal cancer screening tests covered.

Evaluation of this small-media campaign assessed if recipients recalled receiving a postcard from the MCCP and the AHO about getting screened for CRC. A brief 6-question survey was mailed to all recipients 2 weeks after the first postcard was mailed (Group A) and 2 weeks after the second postcard was mailed (Group B). Survey response rates were 46.6% for Group A (234 responses to 504 mailed surveys) and 42.2% for Group B (214 responses to 507 mailed surveys). To assess recall of the small-media campaign, survey respondents were asked, “In the past 3 months, did you receive a postcard in the mail from [name of AHO] and the Montana Department of Public Health and Human Services about getting screened for colorectal cancer?” Respondents also reported demographic information including sex, age group, type of health insurance, and history of CRC screening. The χ^2^ test was used to detect response differences between the 2 intervention groups in univariate analysis. Unadjusted and adjusted odds ratios were calculated by using logistic regression to examine the likelihood that survey respondents would recall or would not recall receiving the reminder postcards, by sex, age group (50–64 years versus 65–75 years), type of health insurance, history of CRC screening, and intervention group. Only those covariates that were statistically significant in the univariate analysis were included in the multivariate logistic regression model. Twelve respondents reported that they were older than age 75; these respondents were excluded from the analysis. Survey data were analyzed by using SAS v 9.3 (SAS Corp, Cary, North Carolina).

## Outcome

As a result of the relationship built through the small-media campaign, the AHO sought additional information on cancer screenings from MCCP to include in AHO’s member newsletter and has been in discussions with MCCP about increasing outreach efforts with additional education for their membership. The AHO stated anecdotally that members reported that polyps were found as a result of getting screened.

Survey respondents in the 2 intervention groups were statistically the same (*P* > .05) with respect to sex and age group (Table 1). More survey respondents in Group B reported having health insurance coverage through their employer than did respondents in Group A (Table 1). Most respondents were female, aged 50 to 64 years, and insured through their employer (Table 1).

**Table 1:** Survey Respondents’ Demographics by Intervention Group, Campaign to Increase Colorectal Cancer Screening, Montana, 2012

Respondent Characteristics	Total % (n)	Group A^a^, % (n)	Group B^a^, % (n)	*P* Value
Total	100.0 (435)	100.0 (228)	100.0 (207)	NA
Female	56.3 (245)	53.5 (122)	59.4 (123)	.43
Age group, y				
50–64	87.8 (382)	86.8 (198)	88.9 (184)	.51
65–75	12.2 (53)	13.2 (30)	11.1 (23)	
Health insurance				
Employer	69.7 (303)	66.2 (151)	73.4 (152)	.05
Spouse or partner	19.8 (86)	21.5 (49)	17.9 (37)	
Other (eg, Medicare, VA)	9.9(43)	12.3 (28)	7.3 (15)	
Don’t know	0.7 (3)	0.0 (0)	1.5 (3)	
Ever screened for colorectal cancer				
Yes	70.3 (306)	70.6 (161)	70.1 (145)	.90
No	29.7 (129)	29.4 (67)	30.0 (62)	

Abbreviation: NA, not applicable; VA, Veterans Affairs.

a Group A and Group B received the same postcard reminding them about colorectal cancer screening. Group B received a second reminder postcard 3 weeks later.

Univariate analysis showed that recall of the small-media campaign was significantly greater among women than men (58.4% compared with 47.3%, respectively; *P* = .05) and greater among Group B respondents (64.3%), who received 2 postcards, than Group A respondents, who received 1 postcard (43.4%) (*P* < .001, Table 2). After adjusting for sex, survey respondents who received 2 postcards (Group B) were 2.3 times as likely to recall having received a postcard than were survey respondents who received 1 postcard (Group A) (Table 2).

**Table 2:** Recall of Receiving Postcard Reminders about Colorectal Cancer Screening Among Enrollees of an Associated Health Organization, by Demographic Characteristics and Intervention Group, Montana, 2012

Respondent Characteristics	Recalled Receiving Postcard, % (n)	*P* Value^a^	Unadjusted Odds Ratio (95% CI)	Adjusted Odds Ratio^b^ (95% CI)
Total	53.3 (232)	NA	NA	NA
Sex				
Male	47.3 (86)	.05	1.0	NA
Female	58.4 (143)		1.6 (1.1–2.3)	NA
Age group, y				
50–64	53.1 (203)	.83	1.0	1.0
65–75	54.7 (29)		1.1 (0.6–1.9)	1.2 (0.6–2.1)
Health insurance				
Employer	55.1 (167)	.20	1.1 (0.7–1.5)	1.0 (0.7–1.5)
Spouse or Partner	52.3 (45)		1.1 (0.6–2.3)	1.0 (0.5–2.1)
Other (eg, Medicare or VA)	46.5 (20)		1.0	1.0
Don’t know	0.0 (0)		1.2 (0.4–3.5)	1.0 (0.4–3.1)
Ever screened for colorectal cancer				
Yes	54.6 (167)	.42	1.2 (0.8–1.8)	1.2 (0.8–1.8)
No	50.4 (65)		1.0	1.0
Intervention group				
Group A: 1 postcard^c^	43.4 (99)	.001	1.0	1.0
Group B: 2 postcard^c^	64.3 (133)		2.3 (1.6–3.4)	2.3 (1.5–3.3)

Abbreviation: CI, confidence interval; VA, Veterans Affairs; NA, not applicable.

a *P* values calculated by χ^2^ test.

b Odds ratios adjusted for sex.

c Group A and Group B received the same postcard reminding them about colorectal cancer screening. Group B received a second reminder postcard 3 weeks later.

## Interpretation

The MCCP considers this collaborative project with an AHO a promising approach to implementing evidence-based colorectal cancer screening interventions. Additionally, there are numerous AHOs and self-insured groups in Montana for future partnerships and outreach on CRC screenings.

Although working with AHOs reached a smaller population than could have been reached by working directly with the larger insurance carriers, the individual relationships the MCCP has built with AHOs has been beneficial. The MCCP had the opportunity to tailor messages and use multiple interventions involving use of small media such as postcards with numerous AHOs, which has allowed the program to reach almost 100,000 Montanans, approximately 10% of Montana’s population, with CRC screening messages.

The MCCP attributes its success in developing a positive relationship with this particular AHO to several factors. The MCCP paid the cost for developing and mailing the postcards, which reduced a cost barrier for the AHO. The cost of printing and postage that MCCP paid totaled less than $3,000. Additionally in Montana, state government agencies, most major insurance carriers, and many AHOs are in the same city, so meeting face-to-face was easy. Face-to-face meetings are particularly important for developing relationships with partners during the initial development phase of this project. The creation of a positive relationship with this AHO also opened doors to other organizations, a common method for business relationships in Montana where word-of-mouth and personal relationships can be productive. Lastly, the MCCP found that the position of the person approached within an organization was important. A quality improvement staff person could easily talk about their HEDIS data and screening improvement methods, whereas a communications staff person was more interested in the wording of the message to members and less interested in the data.

The MCCP will collect insurance claims data over the next year from this AHO to determine whether the long-term goal of increasing CRC screenings in this insured population was achieved. To evaluate member use of CRC screening benefits, the AHO requested claims data from the organization’s insurance carrier on behalf of the MCCP for CRC screenings. However, seeing a trend in CRC screening by using these data will take several years. Additionally, it may be difficult to attribute any trend in use of health benefits to the MCCP postcard campaign without measuring additional short-term and intermediate indicators.

Evaluation of this small-media campaign demonstrated that recall increased with a 2-postcard approach compared with 1 postcard, and the MCCP plans to use a similar postcard campaign with additional small AHOs to remind enrollees about CRC screening. The MCCP will also use these evaluation results to approach again the large insurance carriers in Montana to encourage partnership opportunities and increase CRC screening participation among Montanans with health insurance.
